# Potential mechanisms underlying the association between type II diabetes mellitus and cognitive dysfunction in rats: a link between miRNA-21 and Resveratrol’s neuroprotective action

**DOI:** 10.1007/s11011-022-01035-z

**Published:** 2022-07-04

**Authors:** Norhan S. El-Sayed, Soha Elatrebi, Rasha Said, Heba F. Ibrahim, Eman M. Omar

**Affiliations:** 1 Department of Medical Physiology, Faculty of Medicine, Alexandria University, Alexandria, Egypt; 2 Department of Clinical Pharmacology, Faculty of Medicine, Alexandria University, Alexandria, Egypt; 3 Department of Medical Biochemistry, Faculty of Medicine, Alexandria University, Alexandria, Egypt; 4 Department of Histology and Cell Biology, Faculty of Medicine, Alexandria University, Alexandria, Egypt

**Keywords:** Diabetes Type 2, Resveratrol, MicroRNA 21, Insulin Signaling and memory dysfunction

## Abstract

Cognitive impairment is considered as a typical feature of neurodegenerative diseases in diabetes mellitus (DM). However, the exact link between cognitive dysfunction and diabetes mellitus is still vague. This study aims to investigate some of the mechanisms underlying cognitive impairment that associates diabetes mellitus and insulin resistance. We investigated the role of resveratrol as well on cognitive function in experimentally induced type 2 diabetes highlighting on its influence on the expression of brain miRNA 21. Resveratrol is a naturally occurring, biologically active compound that has numerous significant impacts on the body. Type 2 diabetes mellitus was induced by high fat diet followed a single dose of streptozotocin. Diabetic rats were treated with resveratrol for four weeks. Rats were sacrificed after neurobehavioral testing. Hippocampal tissues were used to assess expression of miRNA 21, GSK and oxidative stress markers. Serum samples were obtained to determine glucose levels, lipid profile and insulin levels. Hippocampal and serum AGEs were measured as well and HOMA IR was calculated. We detected memory impairment and disturbed insulin signaling in diabetic rats. These derangements were reversed by resveratrol treatment partially due to increased expression of miRNA-21. Our study pins the role of miRNA-21 in modulating brain insulin signaling and hence alleviating cognitive dysfunction accompanying diabetes mellitus.

## Introduction

Diabetes mellitus is one of the most serious metabolic disorders in humans worldwide. It is manifested by insulin resistance and disturbed insulin secretion. Various complications may issue due to diabetes, such as cardiovascular disease, kidney failure, retinopathy, and autonomic neuropathy (DeFronzo et al. 2015). Furthermore, diabetes influences the central nervous system, mainly the hippocampus, which is a part of the limbic system that is responsible for cognitive functions. It has been reported that there is a positive correlation between diabetes and dementia (Ahtiluoto et al. 2010). Although diabetes-related cognitive dysfunction appears mild to moderate, it can significantly impede daily functioning and significantly reduce quality of life (Brands et al. 2005). However, it is still difficult to pin down the exact mechanisms underlying cognitive deficits in diabetes and to settle on a specific treatment protocol to improve outcomes. Therefore, the development of new treatment modalities improving cognitive dysfunction is of great interest. Therefore, animal models are useful means to study the molecular mechanisms and the pharmacological therapies for type 2 diabetes mellitus (T2DM). The non-transgenic model of T2Dconsisted of a high-fat diet (HFD) to trigger insulin resistance and glucose intolerance, followed by a low dose of streptozotocin (STZ), which produces moderate -cell damage (Qian et al. 2015). This partial damage leads to reduced secretion of insulin by β-cells, developing in glucose intolerance similar to the human form of T2D.

Undoubtedly, one of the crucial aspects of clinical trials of any drug is its safety. Resveratrol (Res) (3, 5, 30-trihydroxystilbene) is a naturally occurring polyphenol called pytoalexinethat is present in different plant species and is characterized by its low toxicity (Snopek et al. 2018). It has showed a neuroprotective effect on some neurodegenerative diseases as it has antioxidant, anti-inflammatory, antiapoptotic, and anticancer properties (Pallàs et al. 2009). However, despite these data, many aspects related to the molecular mechanism of resveratrol action remain to be clarified.

MicroRNAs (MiRNAs) have gained much attention nowadays due to theiressentialfunction in almost all aspects of the brain, such as neurogenesis, neuronal development, and synaptic plasticity (Goldie and Cairns 2012). MiRNAs are endogenous, short, non-coding RNAs that regulate the responses of different genes. One member of this family is miRNA-21, which is implicated in different CNS pathologies, and many preclinical studies have also shown that miRNA-21 could mitigate brain damage and neurological dysfunction through neurogenesis and angiogenesis (Lopez et al. 2017). Furthermore, miRNAs are entangled in responses to hypoxia and ischemia, and in ischemic tolerance induced by ischemic preconditioning (Lusardi et al. 2010). Accordingly, the articles expand our curiosity to study miRNA-21 in the brain of an HFD-STZ diabetic model and investigate the effect of resveratrol on it.

In this study, we discussed how diabetes affects cognitive function in HFD-STZ-induced T2D in rats. Furthermore, the extent to which resveratrol can improve cognitive function and the expression of certain biochemical markers for therapeutic purposes was assessed, focusing particularly on resveratrol’s effect on the expression of miRNA-21 in the hippocampus.

## Material and methods

### Animals and husbandry

Rats used in this study were purchased from and placed in the department of medical physiology, Faculty of Medicine, Alexandria University, Egypt. Thirty adult male Albino Wistar rats weighing 125–150 gm were used in this study. The rats were housed in groups of five in a cage in an air-conditioned room under standard laboratory conditions. Ten days were given for acclimatization before the study.

### The model of T2D rats

**Fig. 1:**
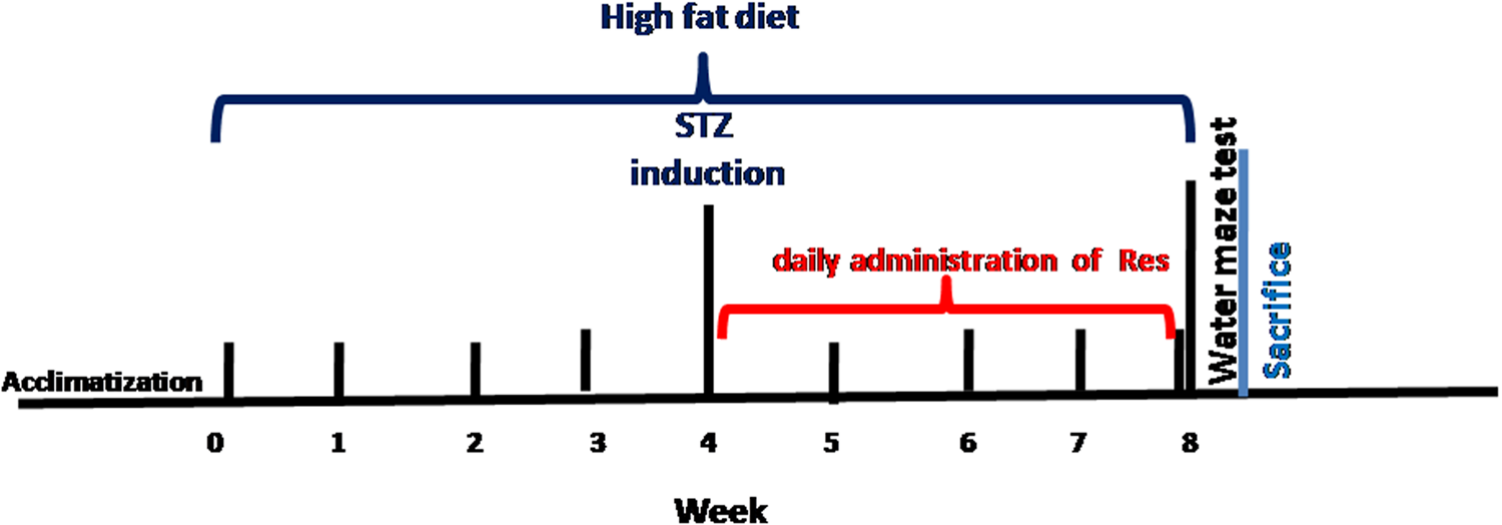
Flow chart for the experimental design of the study. Type 2 diabetes mellitus was induced by high fat diet followed a single dose of streptozotocin. Diabetic rats were treated with resveratrol for four weeks. Afterwards, rats were subjected to neurobehavioral evaluation and then sacrificed for biochemical and histological assessment of the hippocampus

The diabetic model in this study was T2D induced by HFD-STZ (Qian et al. 2015). Diabetes mellitus was induced in 20 rats by intake of HFD (58% fat, 17% carbohydrates and, 25% protein, 310 g/kg butter, 60 g/kg vitamins and minerals, 10 g/kg cholesterol, 1.0 g/kg yeast powder, and 253 g/kg casein and 1.0 g/kg sodium chloride) (Balbaa et al. 2017). It was freshly prepared every week and stored at 4 °C. Diet makes the rat insulin resistant, followed by a single dose of STZ (30 mg/kg)(Sigma Aldrich, St. Louis, MO, USA) dissolved in 0.1 M citrate buffer (pH 4.4) given by intraperitoneal injection after 12 h of fasting. This was followed by administration of a 5% glucose solution to avoid hypoglycemic shock. Blood glucose levels were measured 72 h after STZ injection. Only rats with more than 250 mg/dl were considered to have developed diabetes and were allowed to participate in the study (blood samples were drawn from the tail vein to measure glucose levels using one—touch glucose strips (ACCU-CHEK). Blood sampling was measured four times during the study period (on day zero, 4 weeks after dietary manipulation, 3 days after STZ injection, and at the end of the study). Then diabetic rats were randomly and blindly divided into two groups and continued to be fed on HFD for another four weeks., Fig. 1. During the study period, the general health status of the rats was monitored twice a day, and no side effects were observed.

### Study design and treatment

The study consisted of three groups, with ten rats in each group.** Group I**: Normal control group (ctrl), in which rats were fed with a standard balanced commercial rat diet (10% fat, 70% carbohydrate, and 20% protein) for 8 weeks. **Group II**: Untreated diabetic group (DM) and **group III**: Resveratrol treated diabetic group (DM + Res).The ten treated rats received 99% purity resveratrol (Sigma Aldrich, St. Louis, MO, USA). Resveratrol was prepared freshly in 25% ethanol and was administered intraperitoneally at a dose of 20 mg/kg/day between 10 and 11 a.m. once a day, at a volume not exceeding 0.1 ml / 100 gm of rat weight (Navarro-Cruz et al. 2017, Rašković et al. 2019). Coded vials containing the treatment were prepared by an individual not participating in the experiment to maintain blinding. The treatment continued for four weeks before the training of behavior test.

### Neuro behavioral studies

**Morris Water Maze task** was used to assess the cognitive abilities of all rats**;** the test was performed after four weeks of resveratrol treatment. The maze is a round metal black tank filled with opaque water reaching a depth of 20 cm; a 10 cm in diameter circular black platform was dipped in a fixed location which is 20 cm from the edge and 1 cm below the water surface. The pool was divided into four quadrants: north, south, east, and west. All experimental rats were exposed to four trials of training per day with a hidden platform in one quadrant of the pool for four days. Rats were given 120 s to find the platform, and stayed on the platform for 15 s. If a rat could not find the platform within 120 s, it was guided carefully to the platform and stayed on it for 20 s and its latency time was recorded as 120 s. Memory retention testing was performed 24 h after four days of training. Retention testing entailed 60 s of a free-swimming period with removal of the hidden platform from the pool. The time spent in the target quadrant and the distances swum in the target quadrant were recorded. The percentage of time is calculated as the percent of the time spent in the target quadrant/the total time multiplied by 100, and the percentage of distance is calculated as the distance of swimming in the target quadrant/the total distance of swimming multiplied by 100 (Morris 1981).

### Samples collection

On the last experimental day for each group, 14-h fasting blood samples were collected. Rats were sacrificed by decapitation instantly after behavioral assessments. Up to 3 ml of whole blood was processed for serum isolation, blood samples were taken from the retro-orbital venous plexus of the rat by inserting a capillary haemotocrit tube under light ether anesthesia (Van Herck et al. 1998). Blood was collected into a clean, dry non-heparinized Wassermann tubes for serum separation. The serum was separated by centrifugation at 3000 rpm for 15 min. Aliquots of serum were stored in Eppendorf tubes at -20 °C for measuring biochemical parameters and were not thawed until use. The hemolytic serum samples were excluded. The whole brain was removed, then washed with ice cold saline. The two halves of the hippocampus were separated (Spijker) and stored at a temperature of -80 °C for biochemical analysis.

### Biochemical assessment

#### Determination of some metabolic parameters

The enzymatic colorimetric method is used to measure serum glucose (mg/dl), (Trinder 1969) total serum cholesterol (mg/dl), (Moshides 1987) serum triglyceride (mg/dl) (Fossati and Prencipe 1982) and serum HDL-cholesterol (mg/dl) (Burstein et al. 1970) levels. It was assayed according to the manufacturer's instructions (Bio–diagnostic; Dokki, Giza, Egypt, www.bio-diagnostic.com). Serum insulin levels were measured by using insulin ELISA kit (cat. no. ab273188, WKEA MED SUPPLIES CORP, USA, www.wkeamedsupplies.com). Insulin sensitivity was analyzed using the homeostatic model assessment (HOMA) index (HOMA-IR) = [fasting glucose (mM) x fasting insulin (μU/mL) / 22.5].

#### Determination of hippocampal total antioxidant capacity (TAC) and Malondialdehyde (MDA)

The brain tissues were homogenized in 1–2 ml of 5 mM cold potassium phosphate buffer (pH 7.4), centrifuged at 4000 rpm for 15 min at 4 °C and the supernatant was removed and stored at -80 °C for assay of total antioxidant capacity (Catalog No. TA 25 13, Biodiagnostics) (Koracevic, Koracevic et al. 2001) The determination of the antioxidant capacity is performed by the reaction of antioxidants in the sample with a defined quantity of exogenously supplied hydrogen peroxide. The antioxidants in the sample eliminate a certain quantity of the provided hydrogen peroxide. The residual H_2_O_2_ is detected calorimetrically at 510 nm by an enzymatic reaction that evolves the conversion of 3, 5, dichloer-2-hydroxy benzene sulphonate to a colored product.

The quantitative measurement of the lipid peroxidation marker, MDA, in the brain was performed using a colorimetric assay using TBA reagent. Briefly, 500 μl of brain homogenate supernatant was added up to 1 ml TCA and mixed well, and then the solution was centrifuged at 3000 rpm for 10 min. Then, one milliliter of the supernatant was added to 0.5 ml of TBA, after that, it was boiled for 10 min in a boiling water bath. Finally, the mixture was cooled. The absorbance of samples was read at 532 nm against blank. 1,1,3,3-tetra ethoxy propane (TEP) was used as the reference. The results were normalized to tissue weight to be expressed as ng/gm tissue (Wills 1966).

#### Determination of hippocampalglycogen synthase kinase-3 (GSK-3)by western blotting

Western blot was used to assess GSK-3βexpression in the brain tissue. Brain tissues were homogenized, and the tissue lysate was prepared by adding radioimmunoprecipitation cell lysis (RIPA) buffer (Catalog No. AR0105 boster@bosterbio.com web: http://www.bosterbio.com), Tris (PH 8.0) and protease inhibitor (Catalog No.AR1182, Bosterbio). (25) The lysates then assayed for determination of total protein concentration by the Lowry method and stored until analyzed. After protein electrophoresis, protein transfer from sodium dodecyl sulfate (SDS) polyacrylamide gel onto nitrocellulose membrane was performed by electroblotting. Following a transfer, bands were detected using polyclonal anti-RhoA antibodies (Catalog No.MAB2506 R&D) and anti β-actin antibodies (Catalog No. MAB8929 R &D). Next, the membranes were incubated with the rabbit IgG DAB Chromogenic Reagent Kit (Catalog No. SA2020, Bosterbio). Protein relative band densities ratio were assessed using image J software system and protein expression was normalized against β-actin.

#### Determination of serum and hippocampal advanced glycation end products (AGEs) by ELISA

Frozen brain tissues were homogenized in lysis buffer (150 mM NaCl, 1% Triton X-100 and 10 mM Tris, pH 7.4) containing protease inhibitor, the homogenate was centrifuged at 10,000 g for 10 min at 4 °C and the supernatant was collected. The (Rat AGE) ELISA kit, Cat # STA- 817–5, from Cell Biolabs, USA) used to assay sera and brain AGEs.

#### Determination of hippocampal miRNA-21 by RT-PCR

Hippocampal miRNA-21 was measured by RT-PCR: (Nolan et al. 2006) Total RNA Extraction: Separation of total RNA from serum samples was carried out using the Qiagen® miRNeasy Mini Kit (Qiagen, CA) according to the manufacturer's instructions (Leontariti et al. 2020).

Real-Time Quantitative Reverse Transcription Polymerase Chain Reaction (QRT-PCR): The quantification of miRNA-21 and U6 expression was performed using the Talkman mRNA assay using a two-step RT-PCR.

- Reverse transcription (RT) step: cDNA was reverse transcribed from purified RNA samples by using specific miRNA stem-loop primers from the TaqMan miRNA assays and reagents from the TaqMan® miRNA reverse transcription kit (Applied Biosystem, USA).
- Quantitative real-time PCR (qPCR) step: PCR products were amplified from cDNA samples using the TaqMan miRNA Assays together with the TaqMan® universal PCR master mix (Applied Biosystem, USA). Thermocycling was done using Applied BiosystemsStepOne™ real-time PCR System. The relative expression was calculated using the 2^−∆∆CT^ method.

#### Histological examination of hippocampal tissues

At the end of the experimental periods, the animals were euthanized, and the cerebrum was quickly removed by opening the lateral sides of the skull. Cerebral hemispheres were separated from each other by a medianincision, and the hippocampus was extracted, fixed in 10% buffered formol-saline, dehydrated in ethanol, and embedded in paraffin blocks. Sections of 4–5 μm thickness were cut and stained with hematoxylin & eosin (H&E) for the histological examination.

### Statistical analysis

The mean and standard deviation was used to express all of the data. Statistical analyses were performed with IBM SPSS statistics, version 22.0 (IBM Inc., Armonk, NY, USA). The Kolmogorov- Smirnov was used to verify the normality of distribution of variables, ANOVA was used for comparing the four studied groups and followed by Post Hoc test (Tukey) for pairwise comparison. Pearson coefficient is the correlation between two normally distributed quantitative variables. Significance of the obtained results was judged at the 5% level.

## Results

### Learning and memory assessments in Morris water maze

**Fig. 2:**
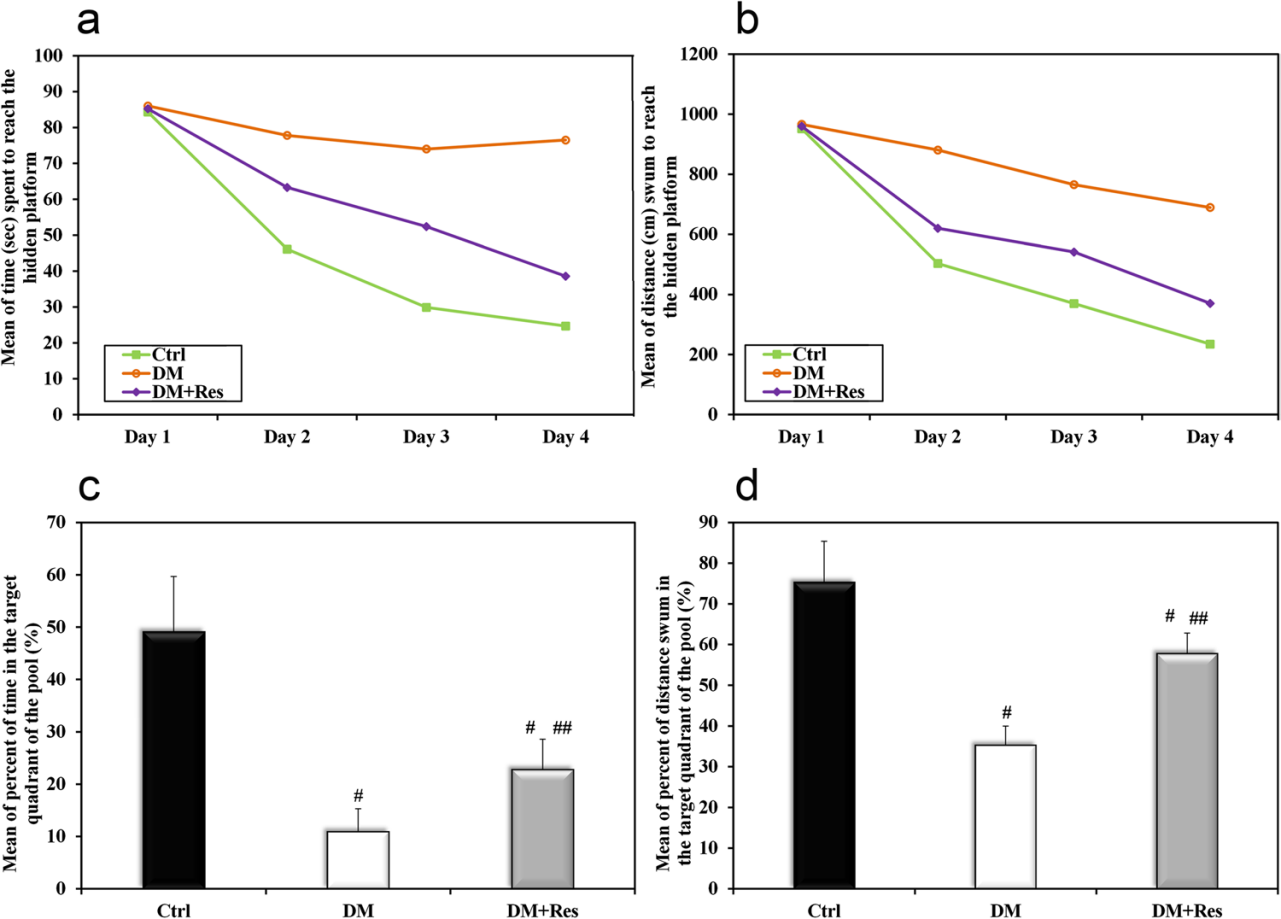
Effect of resveratrol on learning and memory in HFD-STZ T2D rats. **a** Representative tracings of the mean time to reach the hidden platform on the different days of training. **b** The mean distance traveled to reach the hidden platform on each training day. **c** In the memory retention test, the mean percentage of time spent in the target quadrant. **d** In the memory retention test, the mean percentage of distance swum in the target quadrant. Data are presented as means ± SD using one-way ANOVA test (*n* = 10).^#^Significant versus control, ^##^ Significant versus diabetes mellitus, (*p* < 0.05)

The spatial learning in Morris water maze of different experimental groups was represented in Fig. 2(a, b). Mean escape latency (the latency time to locate the submerged platform) and mean escape distance (the path length to locate the submerged platform) was found to be decreasing with training days. In the control group, there was a decrease in the mean of both escape latency and escape distance. Diabetes induction resulted in a significant decline in spatial learning, with longer time and distance to reach the hidden platform, as the difference between the control and untreated diabetic groups was significant in all of training days (*p* < 0.05). These results indicate that diabetes significantly impairs spatial learning and memory in rats. On the other hand, the favorable effect of Res on learning was demonstrated in treated diabetic rats by a significant decrease in the mean time and the mean distance swum to reach the submerged platform compared to those of the untreated diabetic group.

As regards the memory retention test, as shown in Fig. 2(c, d). The normal control rats spent more time and swam more in the target quadrant of the pool, indicating good memory consolidation in this group. However, time spent, and distance swum in the target quadrant was significantly lower in the diabetic group than in the control group. On the other hand, treatment with Res led to an increase in the percentage of time spent and distance swum in the target quadrant to be significantly higher than those in the untreated diabetic group.

### Biochemical assessment

#### Blood glucose level and HOMA –IR

Fasting blood glucose levels of DM group were significantly higher than the control group (> 250 mg/dl throughout the experiment). After the administration of resveratrol for 4 weeks, the fasting blood glucose levels were markedly decreased (173.2 ± 17.3 mg/dl) compared to the DM group (370.6 ± 55 mg/dl) (Table 1).

**Table 1:** Biochemical analysis of the various studied groups

	Control group	Diabetic group	Diabetes + resveratrol group	
Blood glucose level(mg/dl)	101.2 ± 12.2^**b**^	370.6 ± 55^**a**^	173.2 ± 17.3^**ab**^	F = 167.928^*^, *P* < 0.001^*^
HOMA-IR	0.7 ± 0.1^**b**^	3.4 ± 0.6^**a**^	1.5 ± 0.3^**ab**^	F = 145.671^*^, *P* < 0.001^*^
Blood cholesterol level (mg/dl)	65.4 ± 6.7^**b**^	95.8 ± 14.8^**a**^	75.3 ± 17.9^**b**^	F = 12.312^*^, *P* < 0.001^*^
Blood triglyceride level(mg/dl)	93 ± 11.4^**b**^	198.3 ± 15.7^**a**^	111.3 ± 14.1^**ab**^	F = 164.787^*^, *P* < 0.001^*^
Blood HDL- cholesterol(mg/dl)	50.3 ± 3.6^**b**^	22.6 ± 3.3^**a**^	38.4 ± 5.9^**ab**^	F = 97.781^*^, *P* < 0.001^*^

^a^P ≤ 0.05 versus control group, ^b^ P ≤ 0.05 versus diabetic group. Data are expressed as mean ± SD and analyzed by one way ANOVA followed by **Post Hoc Test (Tukey)**. *ANOVA* Analysis of variance, *SD* Standard deviation

**Fig. 3:**
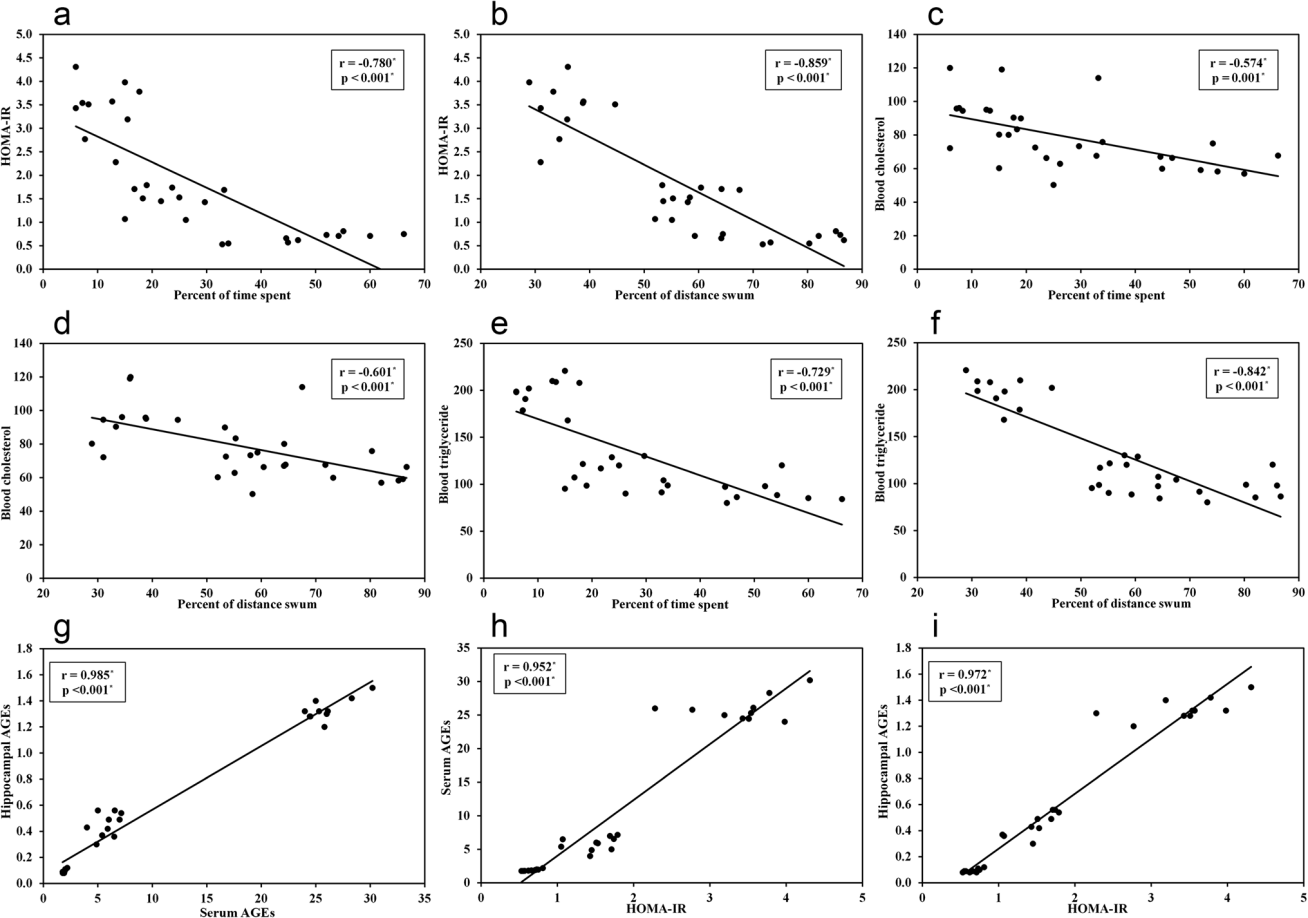
Correlations between different studied parameters. **a** Correlation between HOMA-IR and percent of time spent in target quadrant. **b** Correlation between HOMA-IR and percent of distance swum in target quadrant. **c** Correlation between serum cholesterol level and percent of time spent in target quadrant. **d** Correlation between serum cholesterol level and percent of distance swum in target quadrant. **e** Correlation between serum triglyceride level and percent of time spent in target quadrant. **f** Correlation between serum triglyceride level and percent of distance swum in target quadrant. **g** Correlation between hippocampal AGEs and serum AGEs. **h** Correlation between serum AGEs and HOMA-IR. **i** Correlation between hippocampal AGEs and HOMA-IR

HOMA-IR has been widely used as an insulin resistance index. The diabetic group showed higher HOMA-IR levels (3.4 ± 0.6) compared to the control group (0.7 ± 0.13). However, treatment with Res significantly improved insulin sensitivity. There was a significant decrease in HOMA-IR (1.5 ± 0.3) compared to the normal rats (Table 1).HOMA –IR was found to have a negative correlation with both percent of time and distance in the pool's target quadrant, with r = -0.780, *P* < 0.001 and r = -0.859, *P* < 0.001, respectively, Fig. 3(a, b).

#### Blood lipid profile

Lipid profile was measured for all the rats. The control group showed normal triglycerides (93 ± 11.4 mg/dl), total cholesterol (65.4 ± 6.7 mg/dl) and HDL levels (50.3 ± 3.6 mg/dl). However, there was a marked increase in triglyceride (198.3 ± 15.7 mg/dl) and total cholesterol levels (95.8 ± 14.8 mg/dl) in rats on HFD-STZ (*P* < 0.001) whereas the HDL level (22.6 ± 3.3 mg/dl) was found to decrease (*P* < 0.001) in these animals when compared to controls. Treatment of the HFD-STZ rats with resveratrol daily resulted in a significant decrease in triglycerides (111.3 ± 14.1), total cholesterol (75.3 ± 17.9 mg/dl) and a significant increase in HDL-cholesterol (38.4 ± 5.9 mg/dl) compared to the diabetic untreated group (*P* < 0.001) (Table 1). Serum cholesterol and triglycerides were shown to negatively correlate with the memory retention test. Total cholesterol was found to have a negative correlation with both percent of time and distance in the pool's target quadrant, with r = -0.574, *P* < 0.001 and r = -0.601, *P* < 0.001, respectively, Fig. 3(c, d). A negative correlation was also found between triglycerides and both percent of time and distance in the pool's target quadrant, with r = -0.729, *P* < 0.001 and r = -0.842, *P* < 0.001, respectively, Fig. 3(e, f).

### Hippocampal MDA and hippocampal TAC

**Fig. 4:**
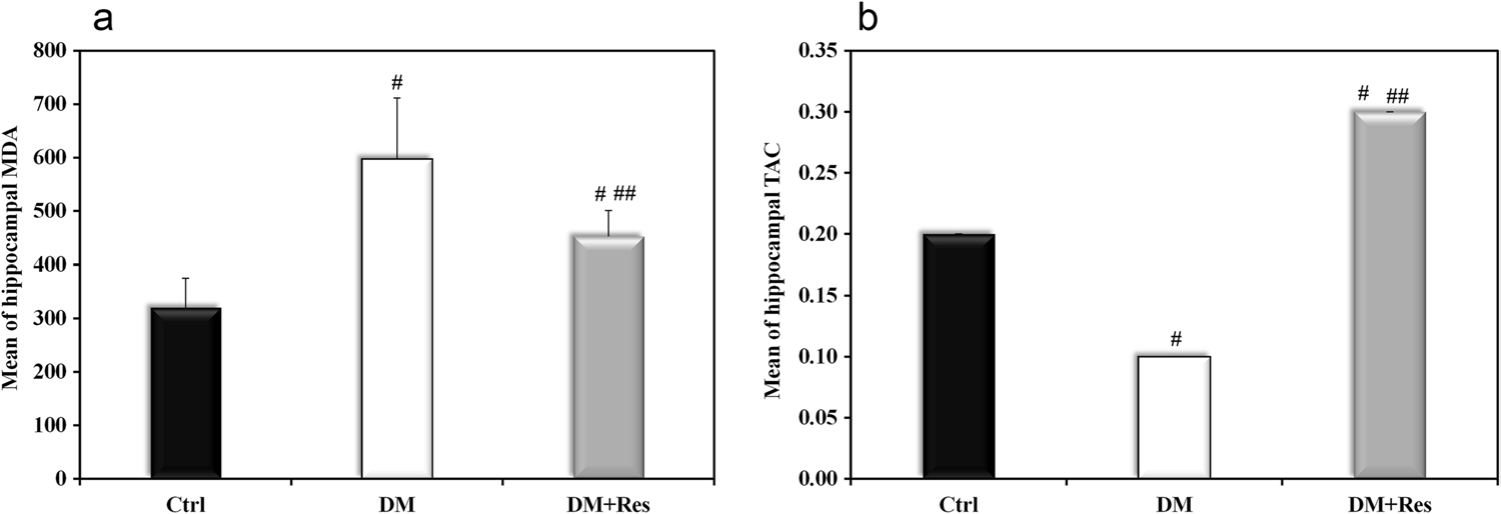
Effect of resveratrol on the oxidative profile in HFD-STZ T2D rats. **a** Graphical presentation of the mean of hippocampal MDA in different studied groups. **b** Graphical presentation of the mean of hippocampal TAC in the different studied groups**.** Data are presented as means ± SD using one-way ANOVA test (*n* = 10).^**#**^Significant versus control, ^**##**^ Significant versus diabetes mellitus, (*p* < 0.05)

The diabetic rats showed a significant increase in MDA levels (597.4 ± 114.3 ng /gm) compared to the control group (320.5 ± 54.4 ng /gm) as well as a significant decrease in TAC (0.1 ± 0 mMol/gm) compared to the control group (0.2 ± 0 mMol/gm); this finding is expected due to STZ cytotoxic effects. The antioxidant effect of resveratrol was also reported in this study by the significant decrease in MDA levels (452.3 ± 48.5 ng /gm protein) and the increase in TAC levels (0.3 ± 0 mMol/gm) (*P* < 0.001), Fig. 4(a, b).

### Protein expression of hippocampus GSK-3βby western blot

**Fig. 5:**
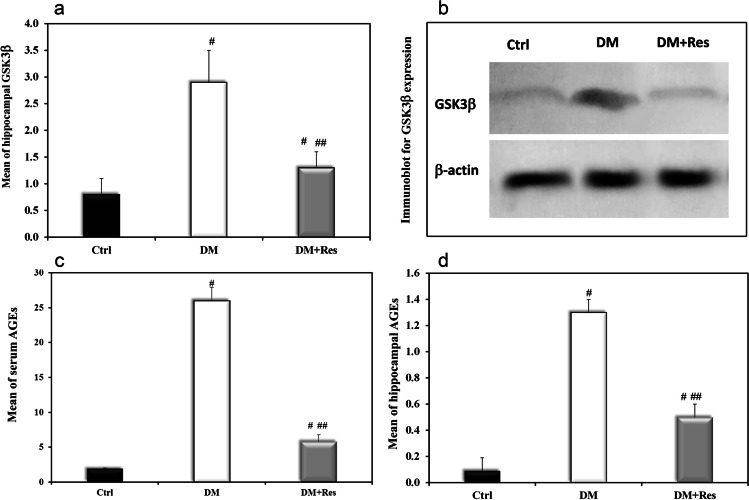
Effect of resveratrol on hippocampal GSK, and serum and hippocampal AGEs in HFD-STZ T2D rats. **a** Graphical presentation of the mean of hippocampal GSK in the different studied groups. **b** Representative immunoblot for the expression of GSK-3. **c** Graphical presentation of the mean of serum AGEs in the different studied groups (**d**) Graphical presentation of the mean of hippocampal AGEs in the different studied groups. Data are presented as means ± SD using one-way ANOVA test (*n* = 10).^**#**^Significant versus control, ^**##**^ Significant versus diabetes mellitus, (*p* < 0.05)

Western blot results showed that the expression of GSK-3β in DM group was significantly higher (2.9 ± 0.6) than in the control group (0.8 ± 0.3) which is considered as evidence of disturbed insulin signaling. However, resveratrol treatment was found to decrease their expression after 4 weeks of administration (1.3 ± 0.3) (*P* < 0.001), Fig. 5(a, b). Hippocampal GSK was positively correlated with HOMA-IR where r = 0.819, *P* < 0.001.

### Serum and hippocampal levels of AGES

**Fig. 6:**
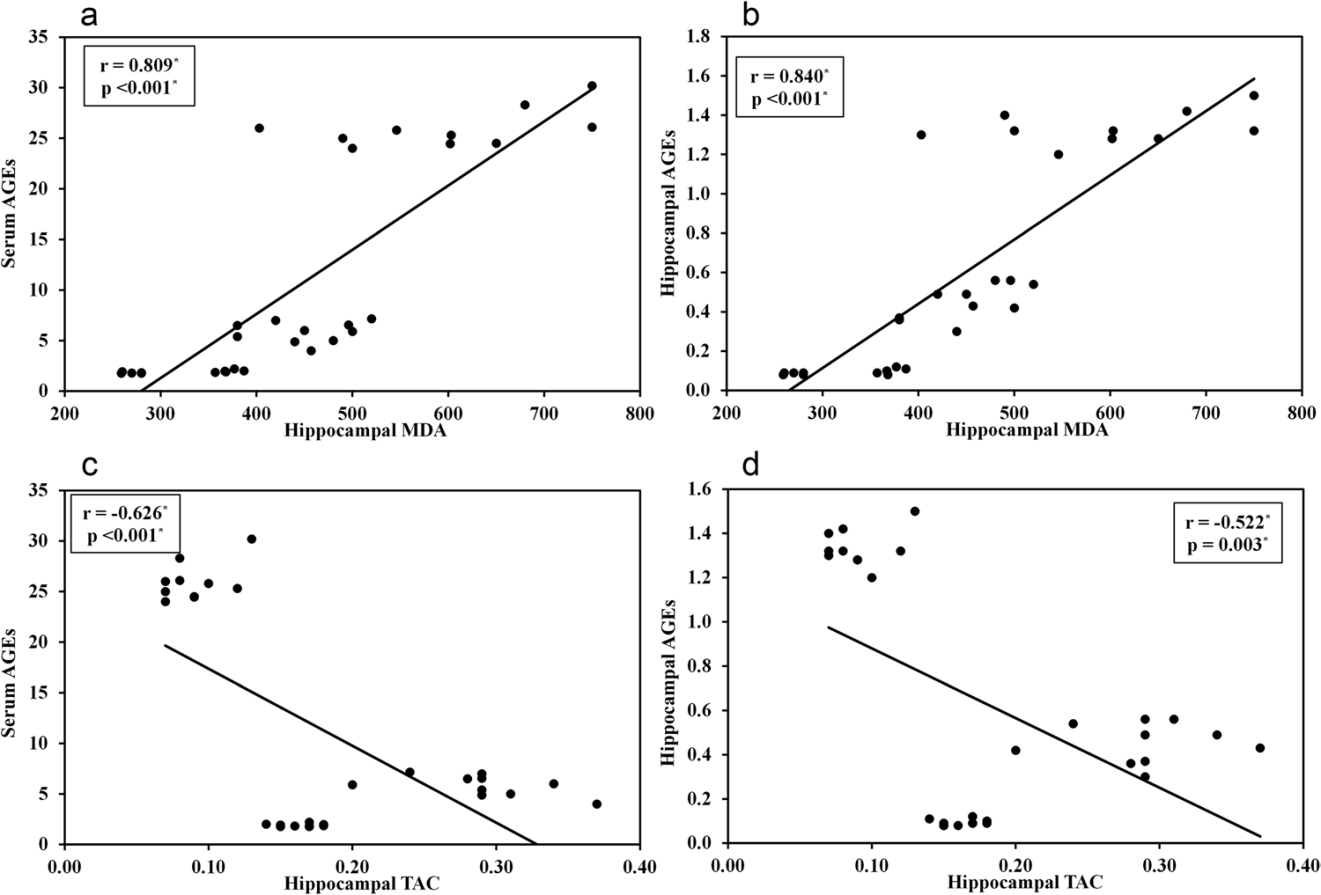
Correlations between different studied parameters. **a** Correlation between serum AGEs and hippocampal MDA. **b** Correlation between hippocampal AGEs and hippocampal MDA. **c** Correlation between serum AGEs and hippocampal TAC. **d** Correlation between hippocampal AGEs and hippocampal TAC

The diabetic group showed higher levels of both serum and hippocampal AGES ((26 ± 1.9 U/ml and 1.3 ± 0.1 U/mg protein respectively) compared to control rats (1.9 ± 0.1 U/ml and 0.09 ± 0.1 U/mg protein). However, resveratrol treatment significantly decreased AGEs levels (5.8 ± 0.1 U/ml and 0.5 ± 0.1 U/mg protein) Fig. 5(c, d). Levels of serum AGEs were positively correlated with hippocampal AGEs where r = 0.985, *P* < 0.001 Figure 3(g). Serum and hippocampal AGEs were positively correlated with HOMA-IR where r = 0.952, *P* < 0.001 and r = 0.972, *P* < 0.001, respectively Figure 3(h, i). Regarding the relation between AGEs and oxidative stress, results showed that serum and hippocampal AGEs were positively correlated with MDA where r = r = 0.809, *P* < 0.001 and r = 0.840, *P* < 0.001, respectively, Fig. 6(a, b). Serum and hippocampal AGEs, on the other hand, were negatively correlated with TAC, with r = -0.626, *P* < 0.001 and r = -0.522, *P* = 0.003, respectively, Fig. 6(c, d).

### Hippocampal miRNA-21

**Fig. 7:**
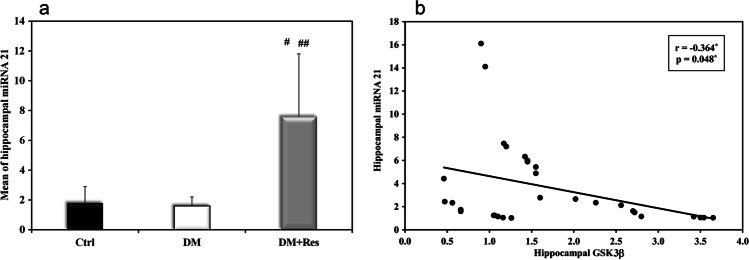
Effect of resveratrol on miRNA-21 in HFD-STZ T2D rats. **a** Graphical presentation of the mean of hippocampal miRNA-21 expression in the different studied group. **b** correlation between hippocampal miRNA-21 expression and hippocampal GSK.Data are presented as means ± SD using one-way ANOVA test (*n* = 10).^**#**^Significant versus control, ^**##**^ Significant versus diabetes mellitus, (*p* < 0.05)

Hippocampal miRNA-21 was significantly over-expressed (7.8 ± 4) in the treated group that received resveratrol in comparison to both the control and diabetic groups. There was no significant difference in the expression of miRNA-21 between the diabetic and control groups, Fig. 7(a). The expression of hippocampal miRNA-21 was negatively correlated with hippocampal GSK-3β where r = -0.364, *P* = 0.048, Fig. 7(b).

### Histological examination of hippocampal tissues

**Fig. 8:**
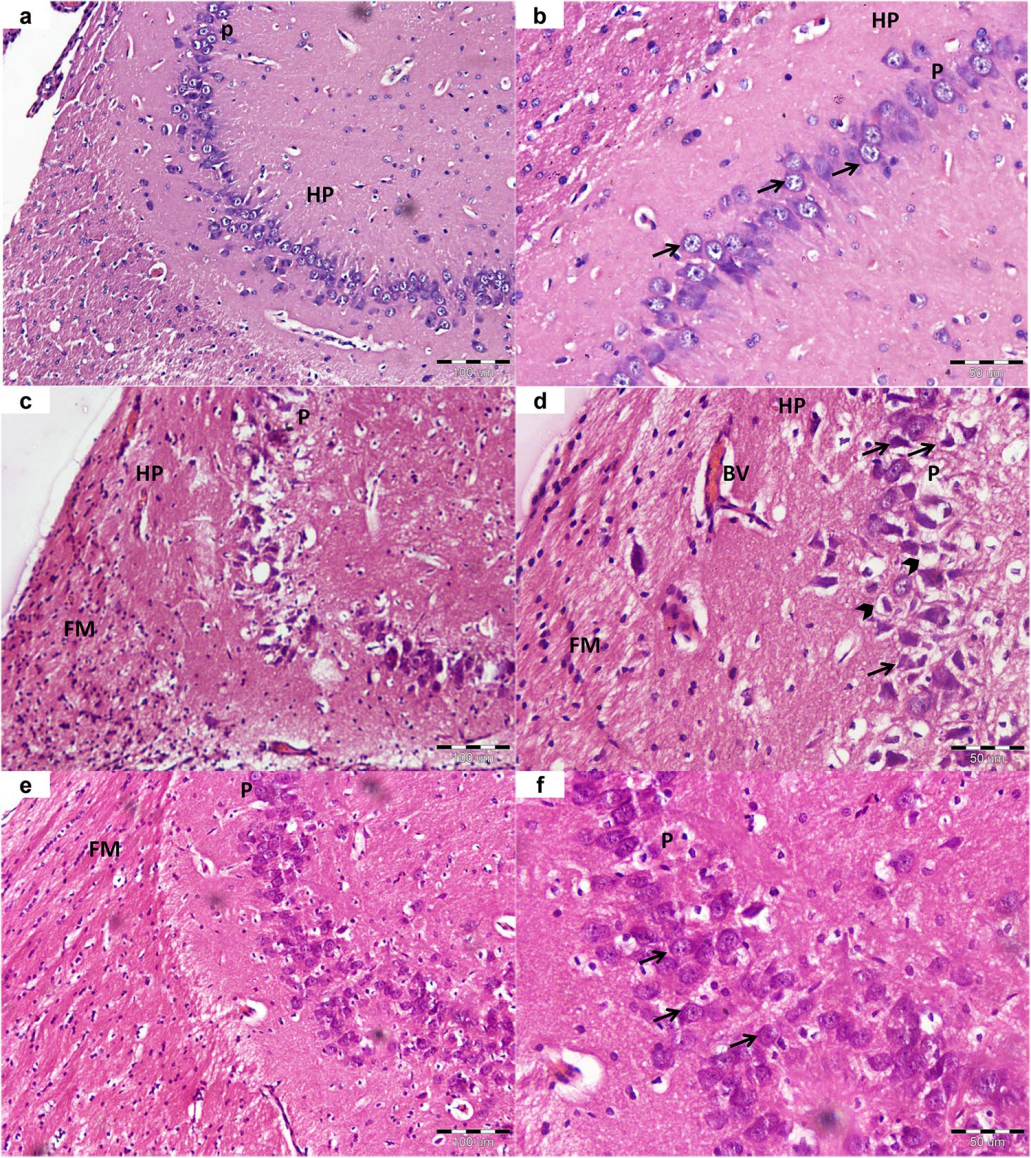
Light photomicrographs of the hippocampus proper of the different studied groups. (H&E stain, Mic. Mag. A, C, E × 200-B, D, F × 400). **a, b** Sections of the control group show a well-organized pyramidal cell layer (P) of the hippocampus proper (HP), formed of closely packed pyramidal cells with large rounded vesicular nuclei, prominent nucleoli and scanty basophilic cytoplasm (arrows). **c, d** Sections of the diabetes group demonstrate marked disorganization and neuronal loss of cells of the pyramidal layer (P). They appear shrunken with darkly stained pyknotic nuclei (arrows) and wide pericellular vacuolization (arrowheads). The hippocampus proper (HP) shows also dilated and congested blood vessels (BV). The fimbria (FM) exhibits vacuolations, hypercellularity, and dissolution of its nerve fibers. **e, f** After treatment with resveratrol, the pyramidal cell layer (P) shows apparently improved thickness and organization. The majority of the neurons appears with vesicular nuclei and prominent nucleoli (arrows) denoting amelioration of the apoptosis. The fimbria (FM) exhibits less vacuolization and cellularity

Light microscopic examination of the hippocampus proper (cornuammonis) of the negative control rats showed the normal architecture of the pyramidal cell layer. The neurons were well-organized with large rounded vesicular nuclei and scanty basophilic cytoplasm, Fig. 8(a, b).

Injection of streptozotocin caused marked degenerative changes affecting the neurons and nerve fibers. The pyramidal cell layer showed disorganization and neuronal loss. Most of the cells appeared shrunken, darkly stained and apoptotic. Extensive pericellular edema and congested blood vessels were detected. The fimbria exhibited vacuolization, dissoluted nerve bundles, and increased cellularity, Fig. 8(c, d).

Except for residual disorganization and edematous changes, the hippocampus proper almost restored the normal appearance of its neurons and nerve fibers after treatment with resveratrol. The pyramidal cells organization were improved, and most of them appeared with large vesicular nuclei and prominent nucleoli, Fig. 8(e, f).

## Discussion

Cognitive impairment is one of the tragic complications of diabetes mellitus, type 2 diabetic patients have an elevated risk for developing Alzheimer’s disease (AD), although the pivotal mechanisms poorly understood. The present study was designed to determine the possible mechanisms of diabetes induced dementia. In addition, this study has investigated the effect of resveratrol on hippocampal miRNA-21 expression and brain insulin signaling as a promising treatment for memory impairments and cognitive dysfunction in diabetic rats.

Several reports have demonstrated that impaired brain glucose utilization and energy metabolism represent early abnormalities that occur before or alongside the onset of cognitive impairment (Cunnane et al. 2011). In both DM and Alzheimer's disease, brain glucose utilization insufficiency and insulin signaling decline are prevalent (Götz et al. 2009).

In the current study, Morris water maze was applied to observe the effects of diabetes on cognitive dysfunction. Untreated diabetic rats showed a significant decline in spatial learning, with longer latency and distance to reach the hidden submerged platform. Moreover, during the memory retention test, rats were not able to remember the exact location of the platform, spending significantly less time in the target quadrant compared to those in the normal group. These results are in line with previous studies that reported similar cognitive derangements in rats suffering from T2DM (Chen et al. 2021). However, this cognitive dysfunction was reversed and spatial memory improved with resveratrol treatment. Although several studies have reported a close association between resveratrol and cognitive performance, the underlying mechanisms remain unclear (Wang et al. 2020). Therefore, in this study, we sought to explore the mechanisms behind the beneficial impacts of resveratrol on learning and memory in T2DM.

In the present work, a combined high fat diet with STZ induced peripheral insulin resistance manifested by hyperglycemia and increased HOMA-IR compared to the control group. These findings confirm the induction of type 2 diabetes. It is well known that persistence of hyperglycemia because of diabetes results in neuronal damage, inflammatory response, and dysfunction of insulin signaling, as well as cognitive impairment (Hamed 2017). Here in our study, resveratrol treatment led to decreased HOMA-IR compared to the control group, indicating amelioration of peripheral insulin resistance. It was reported that defects in insulin signaling in classical peripheral target organs (liver, adipose tissue, and skeletal muscle) are associated as well with disturbed insulin signaling in non-classical organs like the brain, which contribute to the biochemical consequences of insulin resistance (Cardoso and Moreira 2019). This association was detected in our study by reporting a negative correlation between both HOMA-IR and the percent of time and distance spent in the target quadrant of the pool, demonstrating the detrimental effects of insulin resistance on the brain.

In the current study, diabetic rats showed brain insulin resistance, which was justified by an alteration in GSK and a positive correlation between HOMA-IR and hippocampal GSK. This state of brain insulin resistance may be the cause of memory loss in diabetic rats. This was in agreement with several studies that have reported memory disturbances in cases of disturbed brain insulin signaling (El Sayed et al. 2021). Different studies and clinical trials have shown that resveratrol treatment notably lowers blood glucose levels and improves insulin action in type 2 diabetic patients. (Hoseini et al. 2019) However, other studies performed on patients with type 2 diabetes have not confirmed the beneficial effects of resveratrol on blood glucose levels and insulin resistance (Timmers et al. 2016). In the current study, serum glucose levels decreased in the resveratrol-treated group compared to diabetic rats. This hypoglycemic effect of resveratrol was accompanied with decreased serum and hippocampal AGEs levels in resveratrol-treated rats in comparison to diabetic ones. Different studies as well showed increased levels of AGEs in the brains of diabetic rats (Balbaa et al. 2017). On the contrary, other studies showed resveratrol inhibitory effect on AGEs formation in vitro (Maleki et al. 2022).

Poor glycemic control boosts the accumulation of AGEs (Goh and Cooper 2008),their accumulation may lead to molecular and cellular damage (Domínguez et al. 1998), which contributes to brain aging induced by diabetes (Reagan 2002). This was obvious in this study, where a positive correlation was detected between HOMA-IR and both serum and hippocampal AGEs. AGEs are the end products of non-enzymatic reactions with sugar derivatives, that result in irreversible protein cross-links (Oudegeest-Sander et al. 2013). When AGEs are linked to long-lived proteins e.g.; collagen in the arterial wall, they lead to arterial stiffness (Bakris et al. 2004). Furthermore, AGEs bind to specific endothelial AGE-binding receptors and extinguish nitric oxide, thereby leading to endothelial dysfunction (Mashhoody et al. 2014). In diabetes, increased AGEs formation is a likely mechanism of hyperglycemia-induced micro- and macro vascular disease (Engelen et al. 2013). Among other complications of AGEs are their impacts on extracellular matrix proteins and basement membrane components and the formation of protein cross-links that can contribute or facilitate vascular complications (Perantie et al. 2008).

Lipids play an important role in neuronal membrane composition and function. HFD alters the neuronal and vascular components of the brain (Sharma 2021). In the current study, Res treatment nearly normalized different lipid profile parameters of the treated group, where treated rats showed decreased total serum cholesterol and serum TG, and increased HDL in comparison to diabetic rats. The evidence for the effects of resveratrol on dyslipidemia is less conclusive, Rašković A et al. (Rašković et al. 2019) reported that resveratrol significantly improved the lipid profile in dyslipidemicrats induced by a high cholesterol diet and failed to detect this significant difference in diabetic rats induced by high fructose and STZ. This might be explained by the suggested mechanism of action of resveratrol, which includes changes in the expression and activity of enzymes participating in cholesterol metabolism, which require a certain period of time for the full effect to be achieved.The present study supports, to some extent, this explanation. A negative correlation between serum cholesterol, triglyceride, and memory retention test parameters highlighted this lipid-memory crosstalk. This may point to an observation that resveratrol's protective effects against dyslipidemia may be partly behind its neuro-protective effects.

The role of oxidative stress in the pathogenesis of diabetes-induced cognitive dysfunction cannot be overlooked. Oxidative stress consequences and inflammatory response in diabetes-related cognitive impairment has gradually grabbed the attention of researchers (Tian et al. 2016). In the present study, we investigated the oxidation profile by measuring levels of MDA and TAC in the brain. AGEs have been associated with increased oxidative stress and inhibition of reactive oxygen species (ROS) has been found to hinder the formation of AGEs (Nishikawa et al. 2000). The interaction of AGEs with their receptors promotes the production of ROS and activates the protein kinase C (PKC) and nuclear factor-kappa B (NF-κB) (Wendt et al. 2003). Furthermore, ROS may act as a catalyst for the production of more AGEs (Yim et al. 2001). Diabetic rats showed higher oxidative pressure, which was revealed by higher MDA and lower TAC compared to the control group. The combination of oxidative stress and reduced antioxidant defense creates a detrimental combination that disrupts cell functions and damage cells, leading to loss of synapses and cell death (Oyefeso et al. 2021). Therefore, it is reasonable to assume that resveratrol treatment improves brain oxidative balance in T2DM as resveratrol was able to reduce the levels of MDA and increase the levels of TAC. Increased TAC activity acts as a compensating mechanism for oxidative stress and sustained overproduction of free radicals (Aksoy et al. 2003). This interplay between AGEs and oxidative stress was noticeable in this study by their negative correlation with hippocampal MDA and their negative correlation with TAC.These results run in parallel with previous studies that linked the development of amyloid plaque and oxidative stress, together with lipid peroxidation markers such as MDA (Padurariu et al. 2010). Hyperglycemia was also reported to activate various signaling pathways, which led to increased ROS generation and induced insulin resistance (Yu et al. 2006).

Insulin receptors (IR) are co-expressed in specific brain areas, such as the hippocampus. When the IR is activated, the insulin receptor substrate (IRS) recruits a variety of specific molecules, amongst them PI3K, which becomes phosphorylated and therefore activated (Johnston et al. 2003). This, in turn, activates protein kinase B (Akt/PKB). Activated Akt is able to phosphorylate numerous specific substrates that promote glucose transporter type-4 (GLUT4) translocation and glucose uptake, (Sakamoto and Holman 2008) and Glycogen Synthase Kinase-3 (GSK-3), which is a serine/threonine kinase that inhibits glycogen synthase (GS) (Embi et al. 1980). The function of GSK-3 was initially thought to be only phosphorylation and therefore inactivation of GS. However, later studies have shown that GSK-3 can also phosphorylate tau protein (Paudel et al. 1993). Humans have two subtypes of GSK-3: GSK3α and GSK-3β, the latter of which performs a crucial role in tau protein phosphorylation. It was reported that overexpression of GSK-3β promotes abnormal hyperphosphorylation of tau protein, exacerbates neuronal degeneration, disrupts normal synaptic plasticity, and accelerates the pathological process in Alzheimer's disease patients (Hernandez et al. 2013). The current study showed increased hippocampal levels of phosphorylated GSK-3β in diabetic rats compared to the controls, indicating a defect in the insulin signaling pathway in the HFD-STZ model.

Exposure to AGEs has been reported to increase susceptibility to dysmetabolic IR through alteration of insulin receptor, IRS-1, IRS-2 phosphorylation (Cai et al. 2012). This suggests that insulin resistance in the present study may be due to increased accumulation of AGEs in the hippocampus. Resveratrol treatment was found to ameliorate insulin signaling dysfunction through decreasing GSK-3β expression levels. SzkudelskaKet al, reported similar results, where they reported that resveratrol has improved insulin signaling in rats with congenital type 2 diabetes as well (Szkudelska et al. 2021).

In our study, we related resveratrol's effect on the improvement of insulin signaling and subsequent memory improvement to miRNA 21. On examining hippocampal expression levels of miRNA-21, there was no significant difference in its expression levels between the diabetic group and the control group. However, treatment with resveratrol for 21 days led to a significant increase in the expression of miRNA-21 compared to control and diabetic groups.

MiRNAs are new components of insulin signaling machinery which are able to modulate the expression and activity of those factors actively participating in the regulation of insulin signal transduction (Nigi et al. 2018). Accumulating evidence reported and confirmed a correlation between miRNA-21 and PTEN post-transcriptional regulation in several cellular contexts (adipocytes, myotubes and hepatocytes), thus finally attributing an essential role to this miRNA in insulin signaling modulation through the regulation of PTEN phosphatase in a broader set of tissues (Seeger et al. 2014). By diminishing the intracellular levels of PIP3, PTEN hinders the activation of downstream proteins of the PI3K pathway, including the protein kinase C (PKC) and the serine/threonine kinase AKT (Chen et al. 2018). This will, predictably, reduce the expression of phosphorylated GSK-3β, modulating insulin signaling in the brain and decreasing tau protein phosphorylation. A negative correlation was detected between mi-RNA-21 and the insulin signaling parameter GSK-3β. This confirms the beneficial effect of miRNA-21 in improving brain insulin signaling. Therefore, therapies directed towards inducing miRNA-21 may represent a promising strategy in the treatment of diabetes-associated cognitive dysfunction. Undoubtfully, inhibiting the action of miRNA-21 may provide a better apprehension to its exact regulatory mechanism, but unfortunately, this was not assessed in this study. Accordingly, we recommend these investigations in further studies to give more detailed assessment of the role miRNA-21 in the regulation of cognitive function.

## Conclusions

Our study highlighted some of the mechanisms underlying the cognitive impairment that may issue in some diabetic patients. These mechanisms are disturbed lipid profile, peripheral insulin resistance, and increased serum AGEs. Along with these factors, type II diabetes is associated with increased brain oxidative stress, increased hippocampal AGEs, and impaired brain insulin signaling. This interplay of these metabolic factors eventually leads to memory disturbances in diabetic patients. The current study ascertains a neuroprotective role of resveratrolthrough reversing that aforementioned cognitive disturbance. Resveratrol’s neuroprotective effect was partially attributed to the induction of miRNA 21. Consequently, induction of miRNA 21 may have a promising role in prevention and treatment of diabetes mellitus associated memory and learning derangements.

## Data Availability

The data that support the finding of the study are available on request from the corresponding author.

## Abbreviations

AGEs: Advanced glycation end products
GSK-3: Glycogen synthase kinase-3
HFD: High-fat diet
IR: Insulin receptors
MDA: Malondialdehyde
miRNAs: MicroRNAs
Res: Resveratrol
STZ: Streptozotocin
TAC: Total antioxidant capacity
T2DM: Type 2 diabetes mellitus
